# High endoreduplication after drought-related conditions in haploid but not diploid mosses

**DOI:** 10.1093/aob/mcad159

**Published:** 2023-10-12

**Authors:** D Zumel, X Diéguez, O Werner, M C Moreno-Ortiz, J Muñoz, R M Ros

**Affiliations:** Real Jardín Botánico (CSIC), Plaza de Murillo 2, 28014, Madrid, Spain; Real Jardín Botánico (CSIC), Plaza de Murillo 2, 28014, Madrid, Spain; Universidad de Murcia, Facultad de Biología, Departamento de Biología Vegetal, Campus de Espinardo, 30100, Murcia, Spain; Centro Nacional de Biotecnología (CSIC), Departamento de Inmunología y Oncología, 28049 Madrid, Spain; Real Jardín Botánico (CSIC), Plaza de Murillo 2, 28014, Madrid, Spain; Universidad de Murcia, Facultad de Biología, Departamento de Biología Vegetal, Campus de Espinardo, 30100, Murcia, Spain

**Keywords:** Abiotic stress, bryophytes, *Ceratodon*, DNA content, drought, endopolyploidy, endoreduplication

## Abstract

**Background and Aims:**

Endoreduplication, the duplication of the nuclear genome without mitosis, is a common process in plants, especially in angiosperms and mosses. Accumulating evidence supports the relationship between endoreduplication and plastic responses to stress factors. Here, we investigated the level of endoreduplication in *Ceratodon* (Bryophyta), which includes the model organism *Ceratodon purpureus*.

**Methods:**

We used flow cytometry to estimate the DNA content of 294 samples from 67 localities and found three well-defined cytotypes, two haploids and one diploid, the haploids corresponding to *C. purpureus* and *Ceratodon amazonum*, and the diploid to *Ceratodon conicus*, recombination occurring between the former two.

**Key Results:**

The endoreduplication index (EI) was significantly different for each cytotype, being higher in the two haploids. In addition, the EI of the haploids was higher during the hot and dry periods typical of the Mediterranean summer than during spring, whereas the EI of the diploid cytotype did not differ between seasons.

**Conclusions:**

Endopolyploidy may be essential in haploid mosses to buffer periods of drought and to respond rapidly to desiccation events. Our results also suggest that the EI is closely related to the basic ploidy level, but less so to the nuclear DNA content as previously suggested.

## INTRODUCTION

Endopolyploidy, often referred to as somatic polyploidy, is the presence of cells with different ploidy levels in an organism caused by endoreduplication, which involves one or more rounds (i.e. endocycles) of nuclear genome duplication without mitosis (Nagl, 1976; Brodsky and Uryvaeva, 1977; Melaragno *et al.*, 1993). Endopolyploidization can be quantified by the endoreduplication index (EI), which represents the average number of endocycles per cell within a sample (Barow and Meister, 2003). Endoreduplication is important both in cell differentiation (Nagl, 1976; Lee *et al.*, 2009) and in response to the environment, favouring survival under suboptimal conditions (Gendreau *et al.*, 1998; Engelen‐Eigles *et al.*, 2001; Cookson and Granier, 2006; Bhosale *et al.*, 2018). Despite its prevalence in nature, particularly in plants (Bainard and Newmaster, 2010; Leitch and Dodsworth, 2017), the factors that trigger a cell to enter into the endocycle are not well understood. The level of cyclin-dependent kinases (CDKs) is important for endocycle induction (Beemster *et al.*, 2002; Sugimoto-Shirasu and Roberts, 2003), and this can be modulated by hormones and environmental stress (Myers *et al.*, 1990; Ishida *et al.*, 2010; Kitsios and Doonan, 2011).

The effects of endopolyploidy at the cellular level result from the physical changes that cells undergo as they increase their nuclear DNA content, known as ‘nucleotypic effects’. One of these effects is an increase in cell and nuclear size (Jovtchev *et al.*, 2006; Beaulieu *et al.*, 2008). In addition, the increase in DNA content allows for a greater capacity for gene expression (Lee *et al.*, 2009) and metabolism, as the number of organelles such as chloroplasts and mitochondria also increases with cell size (Butterfass, 1983; Bourdon *et al.*, 2012). Ultimately, these changes may enhance the fitness of the entire organism by inducing or repressing the endocycle at specific developmental stages and in specific cell types (Bhosale *et al.*, 2018).

A growing body of evidence highlights the ecological and evolutionary importance of endoreduplication in response to unfavourable conditions. Previous studies have shown that endoreduplication can be plastically regulated by various environmental factors, such as UV-B radiation (Zedek *et al.*, 2020), salt concentration (Ceccarelli *et al.*, 2006) or biotic interactions (Scholes and Paige, 2011; Goga *et al.*, 2018). Finally, high levels of constitutive endoreduplication have been reported in many drought-adapted succulents, supporting its importance in xeric environments (De Rocher *et al.*, 1990).

Endopolyploidy is particularly common in plants probably due to their inability to move when conditions change (Scholes and Paige, 2015). Furthermore, it is often associated with species with small genomes (Nagl, 1976; Barow and Meister, 2003), as the nucleotypic effects can be detrimental in organisms with high nuclear DNA content (Barow, 2006; Scholes and Paige, 2015). Within the embryophytes, it is known only in the gymnosperm *Ginkgo biloba* (Avanzi and Cionini, 1971), and in angiosperms and mosses (Bainard and Newmaster, 2010; Leitch and Dodsworth, 2017; Bainard *et al.*, 2020). Previous studies have mainly examined endoreduplication patterns in angiosperms, where it is widespread. However, mosses typically have less DNA than angiosperms, and their dominant generation is haploid rather than diploid, suggesting that endopolyploidy may play a greater role in compensating for their smaller amount of nuclear DNA (Bainard *et al.*, 2012, 2020). Indeed, this may be particularly important in mosses from xeric regions that are subject to dehydration/rehydration cycles.

In a study of genome size and endopolyploidy evolution across the moss phylogeny, Bainard *et al.* (2020) found no significant correlation between genome size and EI based on a 48-terminal phylogeny, suggesting that there may be other factors acting on EI besides DNA content. Goga *et al.* (2018) recently demonstrated that endoreduplication can be increased in mosses in response to biotic factors, such as allelochemicals secreted by lichens. However, for bryophytes, the relationship between endopolyploidy and abiotic factors has only been suggested, without any supporting data (Bainard *et al.*, 2020).

In this study, we investigated endoreduplication dynamics in field-grown mosses collected at different times of the year to understand the main factors driving EI in geographically and environmentally distant localities. Our hypothesis is that, given the poikilohydric nature of bryophytes and thus their high dependence on free water availability (Proctor, 2009; Vanderpoorten and Goffinet, 2009), we will find higher EI in localities subject to drought, and in haploids rather than in diploids, which in combination would allow plants to become fully physiologically active rapidly after small amounts of water become available during otherwise dry and warm periods. The aims of the present study are therefore (1) to assess the correspondence between basic ploidy level and EI, i.e. whether EI in haploid mosses differs significantly from that in diploids, and (2) to test whether EI correlates with thermopluviometric conditions indicative of drought occurring prior to sampling.

## MATERIAL AND METHODS

### Study group and plant material

The genus *Ceratodon* (Bryophyta, Ditrichaceae) consists in Europe of three confirmed species according to the most recent studies (Nieto‐Lugilde *et al.*, 2018*a*, *b*; Hodgetts *et al.*, 2020): the cosmopolitan *Ceratodon purpureus* (Hedw.) Brid, the recently described *C. amazonum* Nieto-Lugilde, O.Werner, S.F.McDaniel & Ros restricted to southern Spain, and the rare *C. conicus* (Müll.Hal.) Lindb., a species considered of hybrid origin (*C. amazonum* × *C. purpureus*) that forms self-sustaining populations and must therefore be considered a true species. Although these species are morphologically very variable and difficult to distinguish, DNA content allows them to be classified into three well-defined cytotypes: *C. amazonum* has a nuclear genome ~21 % larger than that of *C. purpureus*, while the nuclear genome size of *C. conicus* is equivalent to that of *C. purpureus* and *C. amazonum* combined (Nieto‐Lugilde *et al.*, 2018*a*, *b*). It forms a good group to study how water availability affects endopolyploidy in field-grown mosses.

Between 2020 and 2021, we collected 294 samples of *Ceratodon* from 67 localities in the Iberian Peninsula, covering elevations ranging from 290 to 2750 m a.s.l. Most collections were made during spring (March–May) and summer (June–August) (Supplementary Data, Table S1). *Ceratodon* species occupy a wide variety of habitats, although they share a preference for bare and removed soils in areas exposed to sunlight. The number of samples collected at each locality varied from one to 30 (mode = 4).

*Ceratodon* includes only dioecious species, with males being less vigorous than females (Slate *et al.*, 2017), which may imply varying levels of endoreduplication between the sexes. However, plants of both sexes reach the same stature, and well-developed males are easy to recognize because their stems each end in a globose perigonium. As we randomly selected complete, vigorous gametophores from each collection patch without signs of apex enlargement, we exclude the possibility of having sampled male plants. Additionally, we refrained from selecting gametophores from the marginal areas of the patches to avoid possible boundary effects on moss development. We used five shoots per patch to average the measurement of DNA content and endoreduplication level. A record of collection data, geographical coordinates and ecological notes for each locality is available in Supplementary Data, Table S1. All specimens are kept at herbarium MUB (Departamento de Biología Vegetal, Universidad de Murcia, Spain).

### Flow cytometry of *Ceratodon* gametophores

We used flow cytometry to measure both the basic DNA content and the endoreduplication level of *Ceratodon* patches. For each sample, we chopped the previously selected shoots together with the internal standard *Zea mays* ‘Golden Bantam’ (with an estimated DNA content of 5.43 pg) in 400 μL of LB01 nucleus isolation buffer (Doležel *et al.*, 1989), and filtered the suspension through a 35-μm nylon mesh. We added the fluorochrome propidium iodide and RNAase IIa at a final concentration of 50 μg mL^−1^, and incubated the samples overnight. We then used a Cytomics FC500 flow cytometer (Beckman Coulter Inc., CA, USA) equipped with a laser tuned to 20 mW and operating at 488 nm. Fluorescence intensity was recorded at 575 nm and measured on a logarithmic scale. Kaluza Analysis 2.1 software (Beckman Coulter) was used for data analysis. To eliminate interference from debris particles and to determine the number of nuclei in each peak, polygonal gates were established around the nuclei of interest on the fluorescence versus side scatter scattergram. Genome size was estimated for all samples by calculating the ratio of the mode fluorescence intensity of the moss 1C peak to that of the standard 2C peak. This ratio was then multiplied by the known DNA content of the standard. Based on this calculation, we assigned a cytotype to each sample. We also calculated the percentage of 1C, 2C and 4C cells for each sample, where the C-value is the amount of DNA in a non-replicated genome (Greilhuber *et al.*, 2005). Thus, 1C refers to the basic ploidy level, 2C implies cells that have undergone one round of endoreduplication and 4C refers to cells that have entered the endocycle twice. To calculate EI, we used the method proposed by Barow and Meister (2003), but modified as suggested by Goga *et al.* (2018) for bryophytes.

### Statistical analyses

Before performing statistical tests, we verified the normality and homoscedasticity of the data in R using the *shapiro.test* and *leveneTest* functions from the *stats* (R Core Team, 2021) and *car* (Fox *et al.*, 2020) packages, respectively. To assess mean differences in EI for each cytotype, we performed ANOVA followed by a Bonferroni test in R using the *lm*, *anova* and *pairwise.t.test* functions of the *stats* package (R Core Team, 2021).

We first examined the relationship between EI and seasonality. In the sampled areas of the Iberian Peninsula with Mediterranean climate, spring is characterized by frequent rainfall and mild temperatures, while summer is hot and dry (Ninyerola *et al.*, 2005). Since sampling was done mostly during spring and summer, we compared the mean EI of samples collected in both seasons for each cytotype using unpaired *t*-tests with the *t.test* function from the *stats* package (R Core Team, 2021).

Second, we performed a short-term thermopluviometric analysis to study the effect of temperature and precipitation on EI. We obtained daily temperature and precipitation data from 47 meteorological stations of the Spanish National Meteorological Agency, which are freely available at https://datosclima.es/Aemet2013/DescargaDatos.html. For each locality, we obtained data from the nearest weather station for each of the 15 d prior to sampling, as we considered this period to be representative of the meteorological conditions that occurred prior to plant collection. The air distance between localities and their respective nearest weather stations varied from 1 to 78 km (mean = 21.9 km). We summarized the daily data recorded by the weather stations for the 15-d period into two variables: *Max Temp* and *Days Prec*. *Max Temp* represents the average of the maximum temperatures, while *Days Prec* corresponds to the count of rainy days, regardless of the amount of accumulated rain. For each cytotype, we analysed how EI responds to weather using two linear models: one for each generated variable, using the *lm* function from the *stats* R package (R Core Team, 2021).

For both seasonal and thermopluviometric analyses, we used the median EI of all samples collected at each locality to analyse general trends in endopolyploidy. We acknowledge that plants grown in the field may be exposed to a wide variety of potentially stressful factors that would affect some samples more than others. However, seasonality and meteorology may collectively stimulate or suppress the stress response at each locality, and this variation is hypothetically captured by the median EI.

## RESULTS

**Fig. 1.**
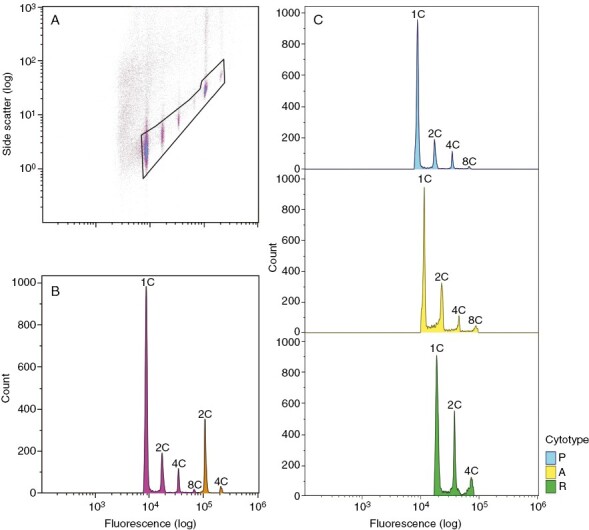
Flow cytometry results. (A) Example of a side scatter versus fluorescence scattergram with a polygonal gate surrounding nuclei of interest to exclude debris particles. (B) Histogram showing the distribution of nuclei counts versus fluorescence intensity, with an example moss of cytotype P (pink) and the standard (orange). (C) Differences in fluorescence intensity among the three cytotypes (P, A and R).

All samples analysed in this study were endopolyploid. Flow cytometry allowed determination of the number of 1C, 2C and 4C nuclei for all mosses. Although we found some samples with 8C nuclei, data above 4C were excluded because the number of nuclei detected at this level of fluorescence was low and in some cases overlapped with debris particles. We identified three cytotypes with distinct fluorescence intensity, which we will refer to as P, A and R (Fig. 1).

**Fig. 2.**
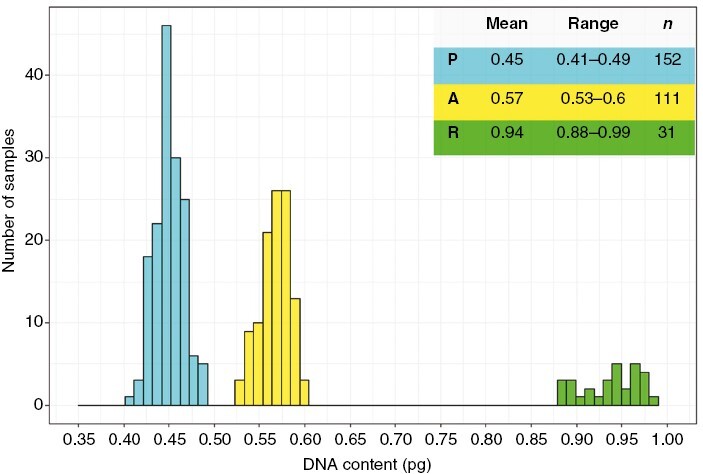
Genome sizes for the 294 samples, showing the differences between the three cytotypes. Mean DNA content and range are given in picograms (pg); *n*, number of samples.

**Fig. 3.**
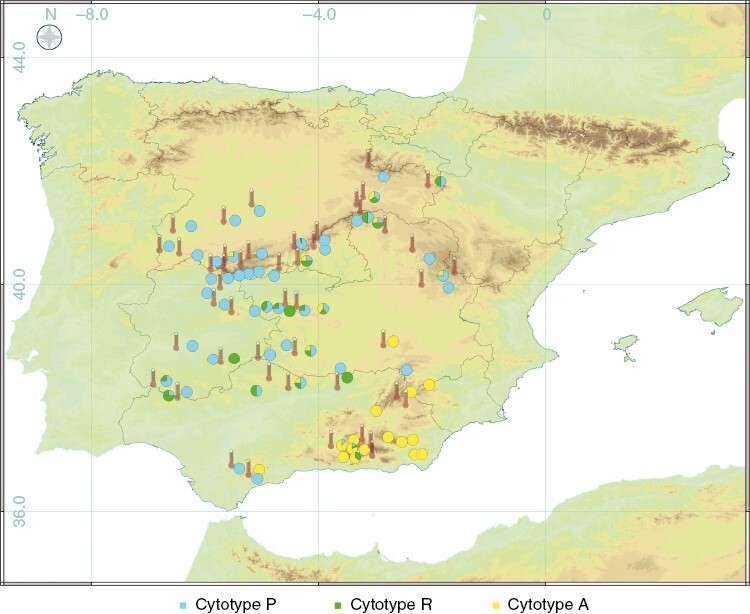
Geographical location of the *Ceratodon* samples included in this study. Pie charts show the proportion of cytotypes found in each of the 67 sampled localities. Thermometer icons indicate weather stations for which data were retrieved for meteorological analysis. Basemap: GTOPO30, doi: 10.5066/F7DF6PQS.

The mean DNA content was 0.45 pg (*n* = 152) in the P cytotype, 0.57 pg (*n* = 111) in the A cytotype and 0.94 pg (*n* = 31) in the R cytotype (Fig. 2). These results are very similar to the DNA content previously reported by Nieto‐Lugilde *et al.* (2018*a*) for the dry material of the haploids *C. purpureus* and *C. amazonum*, and for the recombinant diploid *C. conicus*, respectively. Therefore, we assume that the three cytotypes found correspond to these three species, P and A being haploid, and R being diploid. The P cytotype was found in 50 localities, A in 24 and R in 19. Of the 67 localities sampled, a single cytotype was found in 48, two cytotypes in 12 and all three cytotypes in seven. Of the 12 localities where two cytotypes were found, P and R occurred together in eight, A and P in three, and A and R in one (Fig. 3).

EI was significantly different among the three cytotypes according to Bonferroni’s test (P–A: *P* = 1.5e-11; P–R: *P *= 0.0074; A–R: *P* = 5e-12). Cytotype A had the highest EI (mean = 0.49; s.d. < 0.01), followed by cytotype P (mean = 0.41; s.d. = 0.01) and finally cytotype R (mean = 0.35; s.d. = 0.02). DNA content, assigned cytotype and EI of each sample are listed in Supplementary Data Table S2.

**Fig. 4.**
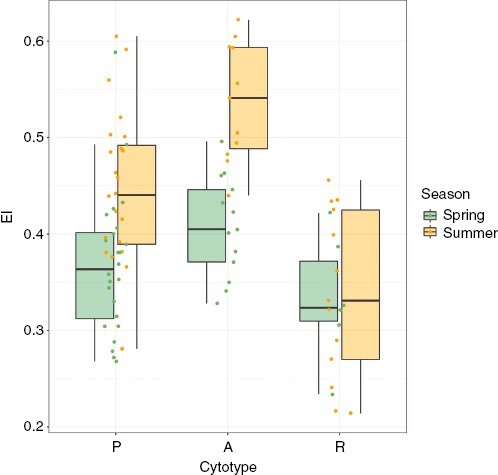
Endoreduplication in spring and summer for each cytotype. Points indicate the median endoreduplication of all samples collected at each locality.

**Fig. 5.**
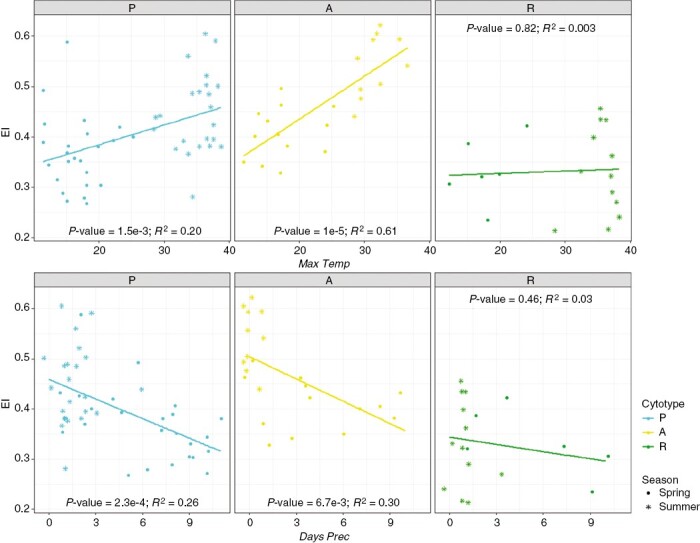
Simple linear regressions between EI and the climatic variables measured during the 15 d prior to collection for each of the three cytotypes. *Max Temp* is the average of maximum temperatures and *Days Prec* is the count of rainy days. Points represent the median EI of all samples collected at each locality.

Regarding seasonal and thermopluviometric analyses, Fig. 4 shows that samples collected in summer have significantly higher EI than those collected in spring for cytotypes P (mean EI of 0.37 in spring vs 0.45 in summer, *P* = 7.5e-4) and A (0.41 vs 0.54, *P* = 1.2e-5), but not for R (0.33 vs 0.34, *P* = 0.9). Simple regressions between EI and the variables *Max Temp* and *Days Prec* showed significantly higher EI in samples collected after hot and dry periods for the P and A cytotypes, but no differences for the R cytotype. Graphs and *R*^2^ values are shown in Fig. 5.

## DISCUSSION

In this study we demonstrate that abiotic stress induces endoreduplication in haploid mosses, but not in closely related but diploid species. Specifically, haploid mosses collected after hot and dry periods, common in Mediterranean summers, have a significantly higher EI than those exposed to colder and rainier conditions, typical of Iberian springs.

Endopolyploidy was significantly different in each cytotype, with higher EI in the haploid cytotypes P and A than in the diploid cytotype R. However, DNA content and EI were not negatively correlated as previously suggested (Nagl, 1976; Barow and Meister, 2003). Although cytotype P has a lower DNA content than cytotype A, its endoreduplication was significantly lower, in agreement with the results reported for mosses by Bainard *et al.* (2020). This suggests that in genomes small enough to avoid the potential negative impact of the nucleotypic effects, EI may not necessarily correlate with genome size. Under stress conditions, plants with haploid genomes would be similarly favoured by increased gene expression and metabolism through endoreduplication (Butterfass, 1983; Lee *et al.*, 2009; Bourdon *et al.*, 2012), regardless of small differences in DNA content. However, this correlation may occur when the difference in genome size implies a difference in the basic ploidy level (as in the R cytotype). Under similar stresses, organisms may tend to endoreduplicate less the more sets of chromosomes they have, since these extra copies would give them a greater capacity to respond to stress without the need for endoreduplication.

Our results reveal considerable variation in EI also at the intraspecific level. The P, A and R cytotypes had an EI range of 0.23–0.73, 0.24–0.74 and 0.19–0.48, respectively. This is particularly important for conceptualizing future research that evaluates endoreduplication dynamics at the macroevolutionary level, as well as for distrusting the results of studies of this type that have already been published. In bryophytes, previous work has evaluated evolutionary trends in endopolyploidy among species from a phylogenetic perspective (Bainard and Newmaster, 2010; Bainard *et al.*, 2020). Although these studies averaged the endopolyploidy estimates of each species from three replicates, these replicates belonged to the same specimen or to mosses from the same population. Thus, the replicates were likely to have experienced the same stressors, which probably caused the standard deviation of the endoreduplication measure per species to be not very high. Both studies state that the samples were collected in the summer of 2009 in Ontario, Canada. Probably, and according to our results, the endoreduplication estimates would have been different if the samples had been collected after different thermopluviometric conditions or in a different season (or after other stressors). Therefore, these data should not be considered representative, and future macroevolutionary studies should look to account for the large intraspecific variability in endoreduplication.

We also found that EI increases during periods of drought in mosses. There is growing evidence linking ploidy plasticity to the ability to respond to suboptimal conditions. Our results are consistent with this research and suggest that haploid mosses in particular may endoreduplicate more to adapt to hot and dry periods. Indeed, the most important stressor for field-grown mosses is water scarcity. Bryophytes are poikilohydric and thus highly dependent on free water, which together with their small haploid genomes may explain why endopolyploidy is the general trend in this group (Vanderpoorten and Goffinet, 2009; Bainard *et al.*, 2020). Mosses are mostly desiccation-tolerant, and cope with drought by suspending physiological activity upon desiccation and increasing energy dissipation through non-photochemical quenching (Beckett *et al.*, 2000, 2005). However, despite the paramount importance of water availability, the relationship between endoreduplication and drought has not been investigated in mosses.

One might think that the EI of plants exposed to periods of drought would be high because plant growth may be slowed under these conditions. Thus, plants collected in spring or after cooler and wetter periods would have more recently formed tissues and therefore have lower EI. Since endoreduplication is an irreversible process (Larkins *et al.*, 2001; Lee *et al.*, 2009), while meristems constantly generate new cells with basic nuclear genomes, endopolyploid cells accumulate as tissues age. Indeed, Paľová *et al.* (2020) realized that the younger (apical) parts of mosses exhibit lower levels of endopolyploidy. Thus, if the growth rate is reduced, the relative proportion of cells with higher ploidy levels, and thus EI, will increase (Joubès and Chevalier, 2000; Paľová *et al.*, 2020). However, our results show that this interpretation may not apply to organisms with non-constant metabolic rates as in the case of the bryophytes studied. *Ceratodon* species are highly desiccation-tolerant mosses (Oliver and Bewley, 1984) that halt their metabolism and thus the entry of their cells into the endocycle when water is not available in the environment (Dhindsa, 1985; Proctor, 2000). Of the 294 plants studied, 292 grew in full sun, where metabolic periods would be shorter due to rapid desiccation compared to mosses growing in shade, which have longer periods of metabolic activity (Hamerlynck *et al.*, 2002). Furthermore, EI was higher after hot and dry periods only in the haploid P and A cytotypes, but not in the R cytotype. The hypothetical accumulation of endopolyploid cells due to non-constant growth in summer or after drought-related conditions would not be evident in the also endopolyploid R cytotype, in which EI shows no relationship with thermotolerance, pluviometry or seasonality. Thus, we suggest that the correlation between EI and thermopluviometric variables may be more closely related to the response to abiotic stress than to the slowing of plant growth.

In angiosperms, endocycle induction stimulated by water shortage can be induced by jasmonate signalling, regulators and transcription factors (Cookson *et al.*, 2006; Harb *et al.*, 2010; Kaplan-Levy *et al.*, 2012; Dubois *et al.*, 2013; Noir *et al.*, 2013). In this biochemically complex and demanding scenario, haploid plants with small genomes, such as mosses, are expected to be more dependent on endoreduplication to compensate for their nuclear DNA deficiency and thus increase gene expression for the activation of cellular mechanisms necessary for drought response. The diploid R cytotype would not be as dependent on this mechanism because it already has two sets of chromosomes, potentially giving it a greater capacity to generate this cellular response.

Endoreduplication may be closely linked to the ability of most mosses to survive desiccation. In previous studies simulating dehydration/rehydration cycles, mitosis was observed to start abnormally late after rehydration (Mansour and Hallet, 1981; Gao *et al.*, 2017). In *Polytrichum formosum*, the first cell division was observed 24 h after water addition, and regular mitotic activity was reached after 48 h, although even after 2 d the proliferative activity was not comparable to pre-drying levels. However, some cells were able to resume DNA synthesis as early as 2 h after rewetting (Mansour and Hallet, 1981). This discrepancy between the M and S phases of the cell cycle may indicate that when mitosis is inhibited, endoreduplication is promoted. Upon rehydration, desiccation-tolerant mosses rapidly resume normal activity (Csintalan *et al.*, 1999). In the moss *Syntrichia ruralis*, RNA and protein synthesis resume within a few minutes after rehydration (Bewley, 1973; Tucker and Bewley, 1976). Thus, in desiccation-tolerant bryophytes, dehydration does not affect nucleic acid metabolism (i.e. DNA replication, transcription and translation), which resumes upon rewetting (Mansour and Hallet, 1981). In Gao *et al.* (2017), the hydration–dehydration–rehydration transcriptomes of the drought-tolerant bryophyte *Bryum argenteum* were analysed. Transcripts related to the stress response were differentially expressed in early dehydration and early rehydration. Some of these transcripts belonged to the transcription factor families AP2/ERF, MYB and HSF. Interestingly, these families have been implicated in endoreduplication and endocycle control (Tominaga *et al.*, 2008; Zhu *et al.*, 2009; Dubois *et al.*, 2013; Rotelli *et al.*, 2019; Ding *et al.*, 2022; Jardinaud *et al.*, 2022; Nomoto *et al.*, 2022). Thus, the association between endopolyploidy and desiccation tolerance seems obvious. Subsequent investigations can empirically validate this, for example by subjecting two sets of cloned, *in vitro*-cultured plants to water stress and to optimal growth conditions, which should be defined, followed by a comparative analysis of the resulting effects. Combining flow cytometry-derived data with gene expression sequencing information can help pinpoint the peaks of endoreduplication during dehydration/rehydration cycles and distinguish differentially abundant transcripts. Furthermore, complementing this approach by quantifying the biomass increase in stressed and control plants over a given period would allow for a comprehensive exploration of the impact of endoreduplication on regeneration dynamics. This investigation would also elucidate whether endoreduplication contributes significantly to an increase in organismal fitness.

## CONCLUSIONS

In this study, we show that abiotic stress influences the level of endopolyploidy in bryophytes. Specifically, we show that endoreduplication in mosses is a plastic process, as we recorded high EI in species that experienced elevated temperatures and low rainfall in the days prior to collection, conditions that occur mainly during summer in the Mediterranean basin. These results suggest that endoreduplication may play a crucial role in the response of bryophytes to drought. However, this relationship occurred only in haploid species, which is consistent with a negative correlation between basic ploidy and endopolyploidy levels rather than between genome size and endopolyploidy levels as previously suggested.

## SUPPLEMENTARY DATA

Supplementary data are available online at https://academic.oup.com/aob and consist of the following.

Table S1: Collection data, ecological notes and geographical information of each locality for which samples were collected for this study. Table S2: DNA content, assigned cytotype and EI of each sample. Cytotype abbreviations are ‘P’ for *Ceratodon purpureus*, ‘A’ for *Ceratodon amazonum* and ‘R’ for the diploid *Ceratodon conicus*.

mcad159_suppl_Supplementary_Tables_S1

mcad159_suppl_Supplementary_Tables_S2
